# Modified extended object tracker for 2D lidar data using random matrix model

**DOI:** 10.1038/s41598-023-32236-w

**Published:** 2023-03-29

**Authors:** Peng Li, Cheng Chen, Cong-zhe You, Jun-da Qiu

**Affiliations:** School of Computer Engineering, Jiangsu University of Technology, Changzhou, 213001 China

**Keywords:** Computer science, Information technology, Aerospace engineering

## Abstract

The random matrix (RM) model is a typical extended object-modeling method that has been widely used in extended object tracking. However, existing RM-based filters usually assume that the measurements follow a Gaussian distribution, which may lead to a decrease in accuracy when the filter is applied to the lidar system. In this paper, a new observation model used to modify an RM smoother by considering the characteristics of 2D LiDAR data is proposed. Simulation results show that the proposed method achieves a better performance than the original RM tracker in a 2D lidar system.

## Introduction

Extended object tracking (EOT) is a major topic in data fusion and has been applied to many different scenarios, including RGB-D sensors^1^, imaging sonar^2^, marine radar^3^, and lidar systems^4^. The extended object may generate more than one measurement per scan, and thus an EOT tracker needs to estimate the kinematic and extended states of the objects concurrently.

The major EOT methods include random hypersurface models (RHMs) and random matrix (RM) models. In RHMs, the kinematical state of an object is updated using a Kalman filter, and the corresponding extended state is described as a given shape model, for example, an ellipsoidal^5^, star-convex^6^, or level-set^7^ model. The kinematic and extended states of an object can be obtained by estimating the parameters of a given shape model. Although an RHM-based tracker can accurately estimate the state of an extended object with different shapes as a complex mathematical model, this approach lacks simplicity and robustness. Compared to an RHM, an RM model is an effective and versatile approach with concise mathematical equations. The RM model is based on the assumptions that “*the densities of the extension state variable are given by inverted Wishart densities*”^8^. The Bayesian recursion characterizes the joint density as a product of Gaussian- and Wishart-related densities with approximate update formula for the parameters characterizing these densities. In addition, the RM model is also known as a Gaussian inverted Wishart (GIW) model.

An RM model used to estimate the state of the group targets was first presented by Koch^8^, and soon after its introduction, numerous studies began focusing on improvements to the model. In^18^, Feldmann et al. proposed an improvement approach that considers the rotation of the object, and it therefore has a faster convergence. A sub-object-based RM model was proposed in^24^. This method uses multiple ellipses to represent a single object, and it can thus track a nonelliptical object. The Bayesian RM filtering and smoothing method was proposed in^16^. An RM method that considers sensor noise and object maneuvers was proposed in^25^. In^26^, a skew-normal-distribution-based RM method was proposed to solve the problem of non-uniform measurements. Moreover, RM models, also known as GIW models, are widely used in multiple extended object tracking, such as GIW-PHD, GIW-GLMB, and Poisson multi-Bernoulli mixture (PMBM) filters^9–12^. For lidar systems, although some RM-based methods have achieved tracking results^9,11,12,17^, the accuracy decreases according to the fact that lidar can only detect one side of an entire object. In^27^, a RM based method was proposed to describe the non-uniformly distributed measurements. In^28^, a learning-based extended object tracking method was proposed for automotive radar. A method combining RM and a virtual measurement model (VMM) was proposed in^29^. VMM is a physics-based function that generates a large number of artificial measurements, and is used to estimate the real extension of an object.

This study proposes a modified RM tracking model suitable for 2D lidar scenarios. The proposed method differs from that described in^29^ and does not generate a large number of artificial measurements. The detection characteristics of the lidar system in the real world are fully considered; thus, the proposed method can better estimate the kinematic and extended states of the object. Note that the proposed method can only deal with single extended object tracking, not to multiple object tracking system. The main contributions of this study are as follows:

- A novel method is proposed to estimate the kinematic vector and extended matrix of the object.
- The proposed method is applied to the Bayesian filtering method described in^16^, which is a robust and effective RM tracker.
- The performances of the proposed and original methods with LiDAR data are compared.

The remainder of this paper is organized as follows. Section "Problem statements" introduces tracking problems. In Section "Modified Rm model for 2D lidar", the RM model is improved based on the detection characteristics of 2D lidar data. Section "Implementation" presents the modified RM tracker for a 2D lidar system. Section "Results" presents the simulation results. Finally, Section "Conclusion" provides some concluding remarks.

## Problem statements

Let $\xi_{k}$ denote the extended state at time *k*: The RM model is usually defined as $\xi_{k} = \left\{ {x_{k} ,X_{k} } \right\}$, where $x_{k}$ is the kinematical vector, $X_{k}$ is an $d \times d$ matrix used to describe the extent of the object, and $d$ is the dimension of the tracking scenario. Using a Bayesian filtering framework, the state prediction is

1 $$ \begin{gathered} p\left( {x_{k} ,X_{k} |Z_{k - 1} } \right) = \iint {p\left( {x_{k} ,X_{k} |x_{k - 1} ,X_{k - 1} } \right)} \hfill \\ \;\;\;\;\;\;\;\;\;\;\;\;\;\;\;\;\;\;\;\;\;\;\;\; \times p\left( {x_{k - 1} ,X_{k - 1} |Z_{k - 1} } \right)dx_{k - 1} dX_{k - 1} \hfill \\ \end{gathered} $$

where $p\left( {x_{k} ,X_{k} |x_{k - 1} ,X_{k - 1} } \right)$ is the density of the transition. The state update is written as

2 $$ \begin{gathered} p\left( {x_{k} ,X_{k} |Z_{k} } \right). \hfill \\ \;\;\;\;\;\;\;\;\;\;\;\; = \frac{{p\left( {Z_{k} |x_{k} ,X_{k} } \right)p\left( {x_{k} ,X_{k} |Z_{k - 1} } \right)}}{{\iint {p\left( {Z_{k} |x_{k} ,X_{k} } \right)p\left( {x_{k} ,X_{k} |Z_{k - 1} } \right)dx_{k} dX_{k} }}}. \hfill \\ \end{gathered} $$

The RM model assumes that $x_{k}$ follows a Gaussian distribution and $X_{k}$ follows an inverse Wishart distribution. Therefore, according to^18^, the object state density based on the factorized model is

3 $$ \begin{gathered} p\left( {x_{k} ,X_{k} |Z_{k} } \right)\; = p\left( {x_{k} |Z_{k} } \right)p\left( {X_{k} |Z_{k} } \right) \hfill \\ \;\;\;\;\;\;\;\;\;\;\;\;\;\;\;\;\;\;\;\; = N\left( {x_{k} |m_{k} ,P_{k} } \right) \times IW\left( {X_{k} |v_{k} ,V_{k} } \right), \hfill \\ \end{gathered} $$

where $N\left( \cdot \right)$ denotes a Gaussian distribution and $IW\left( \cdot \right)$ denotes an inverse Wishart distribution.

**Figure 1:**
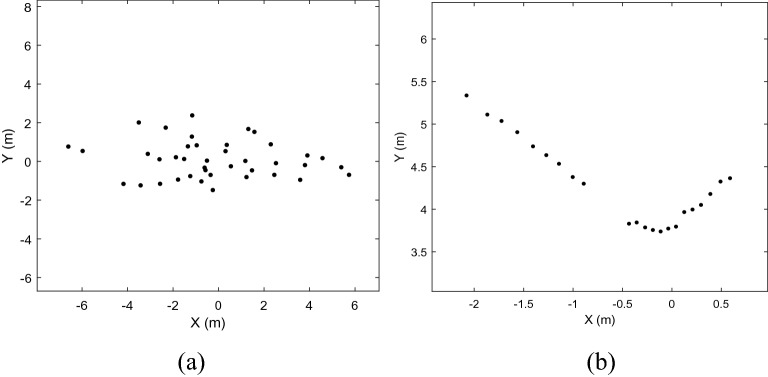
The difference between Gaussian distribution data and Lidar real data. (**a**) Data samples with Gaussian distribution; (**b**) Lidar real data generated by a car.

In the original random matrix model shown in Fig. 1(a), for the measurement set $Z_{k} = \left\{ {z_{k}^{\left( i \right)} } \right\}$, it is typically assumed that each $z_{k}^{\left( i \right)}$ is generated independently from the Gaussian distribution. Therefore, $x_{k}$ and $X_{k}$ are usually estimated using the mean and covariance of the measurement set, $Z_{k}$, respectively. However, in the 2D lidar system, as shown in Fig. 1(b), the measurements are distributed along the contour of the object, and only one side can be detected. The mean and covariance of $Z_{k}$ cannot accurately describe the real position or extent of an object. Therefore, as a problem with the RM model, it cannot estimate the real state of an object when the data are from a 2D lidar system.

In this study, the detection process of the 2D lidar system was analyzed and the RM model was modified by considering the characteristics of the lidar data.

## Modified Rm model for 2D lidar

### Measurement model of 2D lidar

**Figure 2:**
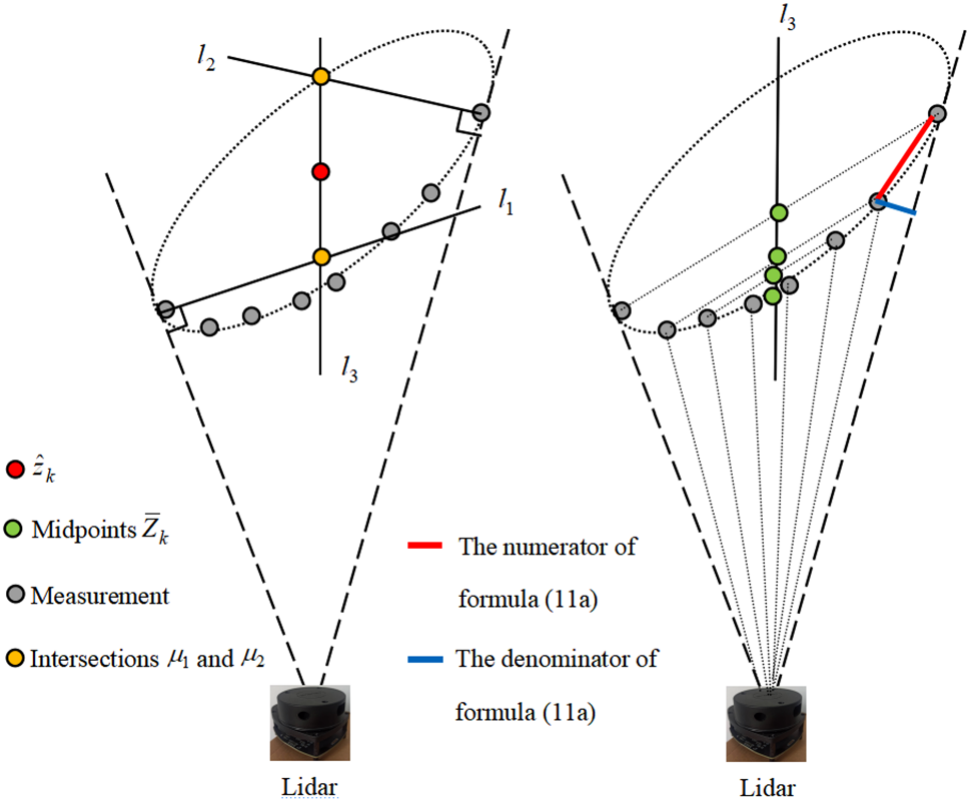
Illumination of the key method.

Raw lidar data are usually in a polar format, for example, $z^{\left( i \right)} = \left\{ {d_{L}^{\left( i \right)} ,r_{L}^{\left( i \right)} } \right\}$, where $d_{L}^{\left( i \right)}$ and $r_{L}^{\left( i \right)}$ denote the distance and radian of the $i$ th ranging per revolution, respectively. According to the detection principle of lidar when applied in the real world (as shown in Fig. 2), the following assumptions were made:

#### *Assumption 1*

The lidar applies a detection $n_{r}$ times per revolution, and the $i$ th observation radian $r_{L}^{\left( i \right)}$ follows a Gaussian distribution $N\left( {{{r_{L}^{\left( i \right)} |i \cdot 2\pi } \mathord{\left/ {\vphantom {{r_{L}^{\left( i \right)} |i \cdot 2\pi } {n_{r} ,\sigma_{r}^{2} }}} \right. \kern-0pt} {n_{r} ,\sigma_{r}^{2} }}} \right)$, where $\sigma_{r}^{2}$ is the given variance.

#### *Assumption 2*

The noise range follows a zero-mean Gaussian distribution with $\sigma_{d}^{2}$ as the variance.

#### *Assumption 3*

According to interference factors, such as weather or strong light, the object has a detection probability of $p_{{D_{1} }}$.

#### *Assumption 4*

If an object can be detected according to the difference in its surface material, the detection probability of each range is $p_{{D_{2} }}$.

Based on the above assumptions, the measurement model can be described as follows:

- Let $Z = \left\{ {z^{\left( i \right)} } \right\}_{i = 1}^{n}$ denote the measurement set per revolution. According to Assumption 3, there is a probability of $1 - p_{{D_{1} }}$ that $Z$ is an empty set.
- When $Z$ is not empty, according to Assumption 4, there is a probability of $1 - p_{{D_{2} }}$ that $z^{\left( i \right)}$ is an empty set.
- Let $\mu_{L}$ and $\hat{z}$ denote the positions of the lidar and object according to Assumptions 1 and 2, where $d_{L}^{\left( i \right)}$ and $r_{L}^{\left( i \right)}$ are defined as follows:
  4a $$ d_{L}^{\left( i \right)} \sim N\left( {c\left( {\hat{z},\mu_{L} ,r_{L}^{\left( i \right)} } \right),\;\sigma_{d}^{2} } \right), $$
  4b $$ r_{L}^{\left( i \right)} \sim N\left( {{{i \cdot 2\pi } \mathord{\left/ {\vphantom {{i \cdot 2\pi } n}} \right. \kern-0pt} n},\;\sigma_{r}^{2} } \right), $$

where $c\left( \cdot \right)$ denotes the detectable contour function of the object. For example, $c\left( {\hat{z},\mu_{L} ,r_{L}^{\left( i \right)} } \right)$ is the ranging result of an object with a center coordinate $\hat{z}$. The coordinates of the lidar is $\mu_{L}$, and the detection radian is $r_{L}^{\left( i \right)}$.

### Proposed method

Let the lidar coordinate system be the center of the coordinate system. Given a measurement set $Z_{k} = \left\{ {z_{k}^{\left( i \right)} } \right\}_{i = 1}^{{n_{k} }}$ in Cartesian coordinates at time *k* (for convenience, polar coordinates are not used), according to Eq. (4), state estimation errors will be caused when using the mean of $Z_{k}$. Assuming that the extent of an object can be approximated as an ellipse, its center $\hat{z}_{k}$ can be approximated as follows:

5 $$ \hat{z}_{k} = {{\left( {\mu_{1} + \mu_{2} } \right)} \mathord{\left/ {\vphantom {{\left( {\mu_{1} + \mu_{2} } \right)} 2}} \right. \kern-0pt} 2}, $$

where $\mu_{1}$ and $\mu_{2}$ are two points on straight line $l_{3}$ passing through the center of the ellipse. According to Assumption 1, two measurements with minimum and maximum radians can be approximated as two tangent points on the tangent line of an ellipse passing through the lidar coordinates. Therefore, the corresponding ellipse tangent equation (i.e., the dotted line in Fig. 2) can be obtained from the coordinates of the two measurement points and the lidar coordinates. As shown in the left half of Fig. 2, $\mu_{1}$ and $\mu_{2}$ can be approximated from the perpendicular tangent of the ellipses $l_{1}$ and $l_{2}$. This is because according to the mathematical properties of an ellipse, $l_{1}$ and $l_{2}$ are the bisectors of the angle formed by the tangent point and focuses of the ellipse. Therefore, the distances from the center to $\mu_{1}$ and $\mu_{2}$ are similar. Note that if the target is small or far from the lidar, the error of (5) will be small. This occurs in this case because the included angle of the two tangent lines is extremely small, and the two straight lines can be regarded as parallel; thus, Eq. (5) is sufficiently accurate.

According to the detection mode of the lidar, $z_{k}^{\left( 1 \right)}$ and $z_{k}^{{\left( {n_{k} } \right)}}$ can be approximated as two tangent points. Suppose that $z_{k}^{\left( 1 \right)} = \left\{ {x_{1} ,y_{1} } \right\}$, $z_{k}^{{\left( {n_{k} } \right)}} = \left\{ {x_{{n_{k} }} ,y_{{n_{k} }} } \right\}$, and $\mu_{L} = \left\{ {x_{L} ,y_{L} } \right\}$. The coordinate equation of the straight-line $l_{1}$ can be obtained as follows:

6a $$ y = A_{1} x + B_{1} , $$

6b $$ B_{1} = y_{1} - A_{1} x_{1} , $$

6c $$ A_{1} = - \frac{{x_{1} - x_{L} }}{{y_{1} - y_{L} }}, $$

and the coordinate equation of the straight line $l_{2}$ is

7a $$ y = A_{2} x + B_{2} , $$

7b $$ B_{2} = y_{{n_{k} }} - A_{2} x_{{n_{k} }} , $$

7c $$ A_{2} = - \frac{{x_{{n_{k} }} - x_{L} }}{{y_{{n_{k} }} - y_{L} }}. $$

The line $l_{3}$ passing through the center of the ellipse can be estimated based on $Z_{k}$. As shown in the right half of Fig. 2, according to Assumption 1, the polar coordinate system can be divided into sectors based on the radian ${{2\pi } \mathord{\left/ {\vphantom {{2\pi } {n_{r} }}} \right. \kern-0pt} {n_{r} }}$, and the number of measurements in each sector may be zero, 1, or higher. By matching the measurements in the corresponding sector, the set $\overline{Z}_{k} = \left\{ {\overline{z}_{i} } \right\}_{i = 1}^{{\overline{n}}}$ of the midpoints can be obtained. Note that if there are multiple measurements in a sector, the mean value of these measurements is used for the calculation. If there are no measurements in a sector, then the measurements in its paired sectors do not participate in the calculation of $\overline{Z}_{k}$. Supposing that $\overline{z}_{i} = \left\{ {\overline{x}_{i} ,\overline{y}_{i} } \right\}$, $l_{3}$ can be fitted using the least square method^19,20^, i.e.,

8a $$ y = A_{3} x + B_{3} , $$

8b $$ \left[ {\begin{array}{*{20}c} {A_{3} } \\ {B_{3} } \\ \end{array} } \right] = \left( {C^{{\text{T}}} C} \right)^{ - 1} C^{T} D, $$

8c $$ C = \left[ {\begin{array}{*{20}c} 1 & \cdots & 1 \\ {\overline{x}_{1} } & \cdots & {\overline{x}_{{\overline{n}}} } \\ \end{array} } \right]^{{\text{T}}} , $$

8d $$ D = \left[ {\begin{array}{*{20}c} {\overline{y}_{1} } & \cdots & {\overline{y}_{{\overline{n}}} } \\ \end{array} } \right]^{T} . $$

The equations for $l_{1}$, $l_{2}$, and $l_{3}$ have been solved; thus, $\mu_{1}$ and $\mu_{2}$ can be obtained by

9a $$ \mu_{1} = \left[ {\frac{{B_{3} - B_{1} }}{{A_{1} - A_{3} }},A_{1} \frac{{B_{3} - B_{1} }}{{A_{1} - A_{3} }} + B_{1} } \right]^{{\text{T}}} , $$

9b $$ \mu_{2} = \left[ {\frac{{B_{3} - B_{2} }}{{A_{2} - A_{3} }},A_{2} \frac{{B_{3} - B_{2} }}{{A_{2} - A_{3} }} + B_{2} } \right]^{{\text{T}}} . $$

Note that if the number $\overline{n}$ is too small, the equation of $l_{3}$ may be inaccurate; thus, a threshold of $\vartheta$ must be given. When $\overline{n} > \vartheta$, $\hat{z}_{k}$ will be obtained using the proposed method, whereas when $\overline{n} \le \vartheta$, $\hat{z}_{k}$ will be solved based on the weighted mean of $Z_{k}$, that is, Eq. (5) should be rewritten as

10 $$ \hat{z}_{k} = \left\{ {\begin{array}{*{20}c} {{{\left( {\mu_{1} + \mu_{2} } \right)} \mathord{\left/ {\vphantom {{\left( {\mu_{1} + \mu_{2} } \right)} {2,\;\overline{n} > \vartheta }}} \right. \kern-0pt} {2,\;\overline{n} > \vartheta }}} \\ {\sum\limits_{i = 1}^{{n_{k} }} {\omega^{\left( i \right)} z_{k}^{\left( i \right)} } \;\;,\;\overline{n} \le \vartheta } \\ \end{array} } \right., $$

where $\omega^{\left( i \right)}$ is the weight of $z_{k}^{\left( i \right)}$. Equation (10) uses the weighted form because the inclination of the object contour affects the measurement density (as shown in Fig. 2). Therefore, $\omega^{\left( i \right)}$ can be calculated as

11a $$ \omega^{\left( i \right)} = \tau \frac{{\left( {z_{k}^{\left( i \right)} - z_{k}^{\left( j \right)} } \right)^{T} \left( {z_{k}^{\left( i \right)} - z_{k}^{\left( j \right)} } \right)}}{{\hat{d}^{2} \left( {i,j} \right)\left( {2 - 2\cos \left( {\left| {r_{L}^{\left( i \right)} - r_{L}^{\left( j \right)} } \right|} \right)} \right)}}, $$

11b $$ \hat{d}\left( {i,j} \right) = \min \left( {d_{L}^{\left( i \right)} ,d_{L}^{\left( j \right)} } \right), $$

11c $$ \tau = \frac{{n_{k} }}{{\sum\limits_{i = 1}^{{n_{k} }} {\omega^{\left( i \right)} } }}, $$

where $z_{k}^{\left( j \right)}$ denotes the measurement closest to $z_{k}^{\left( i \right)}$; $\left\{ {d_{L}^{\left( i \right)} ,r_{L}^{\left( i \right)} } \right\}$ and $\left\{ {d_{L}^{\left( j \right)} ,r_{L}^{\left( j \right)} } \right\}$ are the distance and radians of the $i$ th and $j$ th measurements, respectively; and $\tau$ denotes normalization. In fact, the numerator of Eq. (11a) represents the length of the red line in Fig. 2, whereas the denominator is the length of the blue line. By weighting Eq. (11), the influence of the object contour inclination on the statistical accuracy can be reduced. Similarly, the covariance matrix $\hat{Y}$ of the measurement set $Z_{k}$ can be obtained as

12 $$ \hat{Z} = \sum\limits_{i = 1}^{{n_{k} }} {\omega^{\left( i \right)} \left( {z_{k}^{\left( i \right)} - \hat{z}_{k} } \right)\left( {z_{k}^{\left( i \right)} - \hat{z}_{k} } \right)^{{\text{T}}} } . $$

## Implementation

In this section, the proposed method is used to modify the advanced RM tracker presented in^16^. The tracker comprises three steps: prediction, updating, and smoothing. Let function $M\left( {\text{x}} \right)$ denote the change mode of the object extent using factorized Gaussian inverse Wishart densities^18^. If $M\left( {\text{x}} \right)$ is a given $d \times d$ invertible matrix $A$, the prediction steps are as follows.

13a $$ m_{k|k - 1} = f_{{k{ - }1}} \left( {m_{k - 1|k - 1} } \right), $$

13b $$ P_{{k|k{ - }1}} = \tilde{F}_{{k{ - }1}} P_{{k|k{ - }1}} \tilde{F}_{k - 1}^{T} + Q_{{k{ - }1}} , $$

13c $$ v_{k|k - 1} = d + 1 + \frac{{n_{k - 1} \left( {v_{k - 1|k - 1} - d - 1} \right)}}{{n_{k - 1} + v_{k - 1|k - 1} - 2d - 2}}, $$

13d $$ V_{k|k - 1} = \left( {1 + \frac{{v_{k - 1|k - 1} - d - 1}}{n - d - 1}} \right)^{ - 1} AV_{k - 1|k - 1} A^{{\text{T}}} , $$

where $m_{k + 1|k}$ and $P_{k + 1|k}$ are the motion state and corresponding covariance, respectively; $V_{k + 1|k}$ and $v_{k + 1|k}$ are the Wishart scale matrix and degree of freedom, respectively; $f_{k} \left( \cdot \right)$ and $\tilde{F}_{k}$ are the motion model and corresponding state transition matrix, respectively; and $Q_{k}$ denotes the process noise covariance. If $M\left( {\text{x}} \right)$ is not a given matrix, formulas (13c) and (13d) become:

14a $$ v_{k|k - 1} = d + 1 + \eta^{ - 1} \left( {v_{k - 1|k - 1} - d - 1} \right), $$

14b $$ V_{k|k - 1} = \eta^{ - 1} \left( {1 - \frac{d + 1}{s}} \right)\left( {1 - \frac{d + 1}{{n_{k - 1} }}} \right)C_{2} , $$

14c $$ \eta = 1 + \left( {v_{k - 1|k - 1} - 2d - 2} \right)\left( {\frac{1}{s} + \frac{1}{{n_{k - 1} }} - \frac{d + 1}{{sn_{k - 1} }}} \right), $$

14d $$ s = \frac{d + 1}{d}{\text{Tr}}\left\{ {{\text{C}}_{{1}} {\text{C}}_{{2}} \left( {{\text{C}}_{{1}} {\text{C}}_{{2}} - {\text{I}}_{d} } \right)^{ - 1} } \right\}, $$

14e $$ {\text{C}}_{{1}} = {\text{E}}_{k - 1|k - 1} \left[ {\left( {M\left( {\text{x}} \right)V_{k - 1|k - 1} M^{{\text{T}}} \left( {\text{x}} \right)} \right)^{ - 1} } \right], $$

14f $$ {\text{C}}_{{2}} = {\text{E}}_{k - 1|k - 1} \left[ {M\left( {\text{x}} \right)V_{k - 1|k - 1} M^{{\text{T}}} \left( {\text{x}} \right)} \right], $$

where ${\text{Tr}}\left\{ \cdot \right\}$ is the trace of a matrix, and ${\text{E}}_{k - 1|k - 1} \left[ \cdot \right]$ is is the expectation.

The state updating steps are as follows:

15a $$ m_{k|k} = m_{k|k - 1} + K\varepsilon , $$

15b $$ P_{k|k} = P_{k|k - 1} + KSK^{{\text{T}}} , $$

15c $$ v_{k|k} = v_{k|k - 1} + n_{k} , $$

15d $$ V_{k|k} = V_{k|k - 1} + \hat{N} + \hat{Y}, $$

where

16a $$ \hat{N} = \hat{X}^{\frac{1}{2}} S^{{ - \frac{1}{2}}} \varepsilon \varepsilon^{T} \left( {S^{{ - \frac{1}{2}}} } \right)^{{\text{T}}} \left( {\hat{X}^{\frac{1}{2}} } \right)^{{\text{T}}} , $$

16b $$ \hat{Y} = \hat{X}^{\frac{1}{2}} Y^{{ - \frac{1}{2}}} \hat{Z}\left( {Y^{{ - \frac{1}{2}}} } \right)^{{\text{T}}} \left( {\hat{X}^{\frac{1}{2}} } \right)^{{\text{T}}} , $$

16a $$ Y = \kappa \hat{X} + R, $$

16c $$  S = \tilde{H}P_{{k|k - 1}} \tilde{H}^{T}  + \frac{Y}{{n_{k} }}  $$

16d $$ K = P_{k|k - 1} \tilde{H}^{T} S^{ - 1} , $$

16e $$ \varepsilon = \hat{z} - \tilde{H}m_{k|k - 1} . $$

Here, $\kappa$ is a given scale parameter, and $R$ is the object observation noise. In addition, $\hat{z}$ and $\hat{Z}$ can be calculated using the proposed method, that is, Eqs. (10) and (12).

Note that this study did not contribute to the smoothing steps. Therefore, the smoothing steps are the same as those for the original smoother and can be found in Section V of ^16^.

## Results

This section presents the simulation results of the proposed method and the original approach presented in^16^. There are three scenarios: an ellipse object scenario, a rectangular object scenario, and 2D lidar data scenario. Note that the first two scenarios are based on simulation, whereas the last one uses a real dataset. The lidar parameters are as follows: 3irobotix-3i-T1 2D Lidar, 20-m detection radius, 1800 detections per revolution, and a scan interval of T = 0.1 s. The motion model is a coordinated turn model, that is, the object motion state is $x_{k} = \left[ {{\text{x}}_{k} ,{\text{y}}_{k} ,v_{k}^{{\text{x}}} ,v_{k}^{{\text{y}}} ,\theta_{k} } \right]^{{\text{T}}}$ and the other parameters are as follows:

17a $$ f\left( {x_{k} } \right) = \left[ {\begin{array}{*{20}c} 1 & 0 & {\frac{{\sin \left( {T\theta_{k} } \right)}}{{\theta_{k} }}} & { - \frac{{1 - \cos \left( {T\theta_{k} } \right)}}{{\theta_{k} }}} & 0 \\ 0 & 1 & {\frac{{1 - \cos \left( {T\theta_{k} } \right)}}{{\theta_{k} }}} & {\frac{{\sin \left( {T\theta_{k} } \right)}}{{\theta_{k} }}} & 0 \\ 0 & 0 & {\cos \left( {T\theta_{k} } \right)} & { - \sin \left( {T\theta_{k} } \right)} & 0 \\ 0 & 0 & {\sin \left( {T\theta_{k} } \right)} & {\cos \left( {T\theta_{k} } \right)} & 0 \\ 0 & 0 & 0 & 0 & 1 \\ \end{array} } \right]x_{k} , $$

17b $$ Q_{k} = G{\text{diag}}\left( {\left[ {{0}{\text{.1}}^{{2}} {,0}{\text{.1}}^{{2}} {,}\left( {{\pi \mathord{\left/ {\vphantom {\pi {{180}}}} \right. \kern-0pt} {{180}}}} \right)^{2} } \right]} \right)G^{{\text{T}}} , $$

17c $$ Q_{k} = \left[ {\begin{array}{*{20}c} {\frac{{T^{2} }}{2}{\text{I}}_{2} } & {{\text{O}}_{2 \times 1} } \\ {T{\text{I}}_{2} } & {{\text{O}}_{2 \times 1} } \\ {{\text{O}}_{1 \times 2} } & 1 \\ \end{array} } \right], $$

17d $$ M\left( {\text{x}} \right) = \left[ {\begin{array}{*{20}c} {\cos \left( {T\theta } \right)} & { - \sin \left( {T\theta } \right)} \\ {\sin \left( {T\theta } \right)} & {\cos \left( {T\theta } \right)} \\ \end{array} } \right], $$

17e $$ \tilde{H}_{k} = \left[ {\begin{array}{*{20}c} {{\text{I}}_{2} } & {{\text{O}}_{{{2} \times {3}}} } \\ \end{array} } \right], $$

where the lidar scan interval is $T = 1$ s. In addition, ${\text{I}}$ and ${\text{O}}$ denote the identity matrix and zero matrix, respectively.

Measurements were generated using the model proposed in Section "Measurement model of 2D lidar". The detection probability of the object was $p_{{D_{1} }} = 0.9$, and the detection probability of each range was $p_{{D_{2} }} = 0.9$. The Lidar conducts a detection 7200 times per revolution, and the observation noise is $\left\{ {\sigma_{d} = 0.1^{2} ,\sigma_{r} = 0.001^{2} } \right\}$.

The performances of different methods are judged based on the Gaussian Wassterstein distance (GWD) metric^21^^-^^22^, which considers both the object location and extension errors, and is defined as follows:

18 $$ \begin{aligned} \Delta_{\left. k \right|l} & = \left( {\left\| {{\text{p}}_{k} - {\hat{\text{p}}}_{\left. k \right|l} } \right\|} \right.^{2} \\ & \quad \left. { + {\text{Tr}}\left( {X_{k} + \hat{X}_{\left. k \right|l} - 2\left( {X_{k}^{{\tfrac{1}{2}}} \hat{X}_{\left. k \right|l} X_{k}^{{\tfrac{1}{2}}} } \right)^{{\tfrac{1}{2}}} } \right)} \right)^{\frac{1}{2}} , \\ \end{aligned} $$

where $\Delta_{k|l}$ is the GWD value at time *k*. The first half describes the square of the location error, and the second half describes the square of the extension error. The GWD metric is used to indicate the similarity of the two distributions, that is, the smaller the value $\Delta_{k|l}$, the more similar the two distributions are.

### Ellipse object scenario

In this section, the real shape of the object is an ellipse.

**Figure 3:**
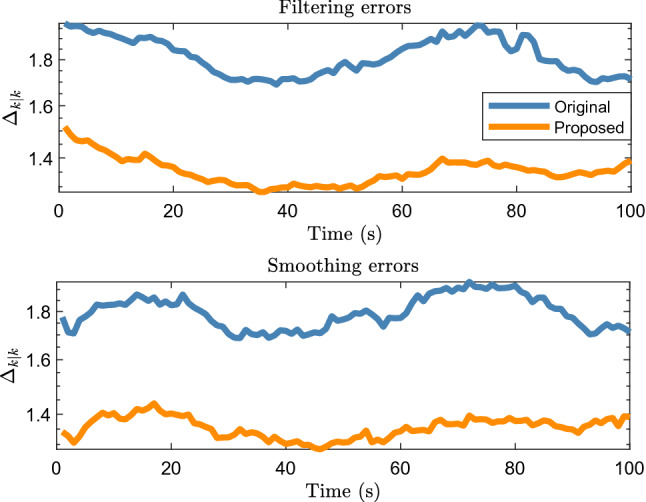
Average filtering and smoothing results of elliptical object.

Figure 3 shows the filtering and smoothing results of 200 Monte Carlo runs. The GWD errors of the proposed method are lower than those of the original method, which shows that the proposed method improves the tracking accuracy.

**Figure 4:**
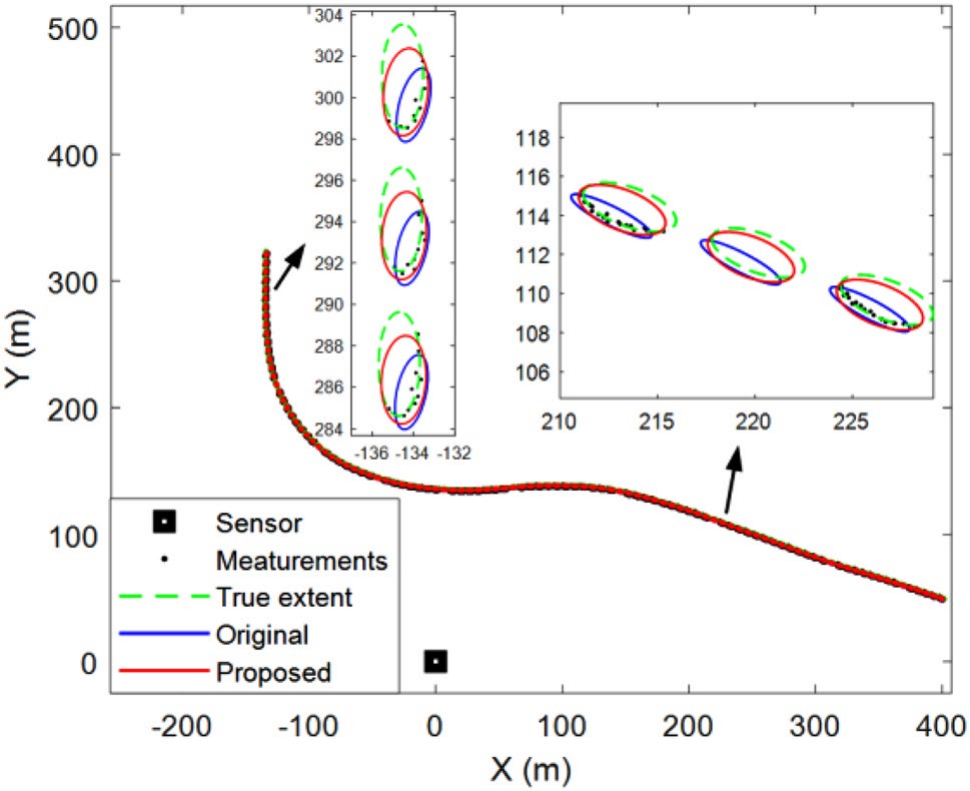
Elliptical object tracking results of a single run.

Figure 4 shows the tracking results for a single run. It can be seen intuitively that, compared with the original method, the object motion and extent states estimated by the proposed method are closer to the real situation, which is also the reason for the difference in the errors in Fig. 3.

**Figure 5:**
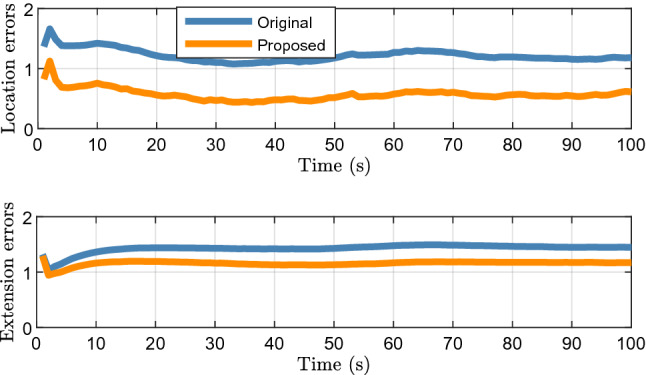
Average location errors and extension errors of elliptical object.

Figure 5 shows the average location and extension errors of 200 Monte Carlo runs. It can be seen that the difference between the location errors of the proposed method and the original filter is greater than the extension errors. This shows that the method proposed in Section "Modified Rm model for 2D lidar" can effectively estimate the location of the target center, which is the main reason for the improvement in the tracking performance.

**Figure 6:**
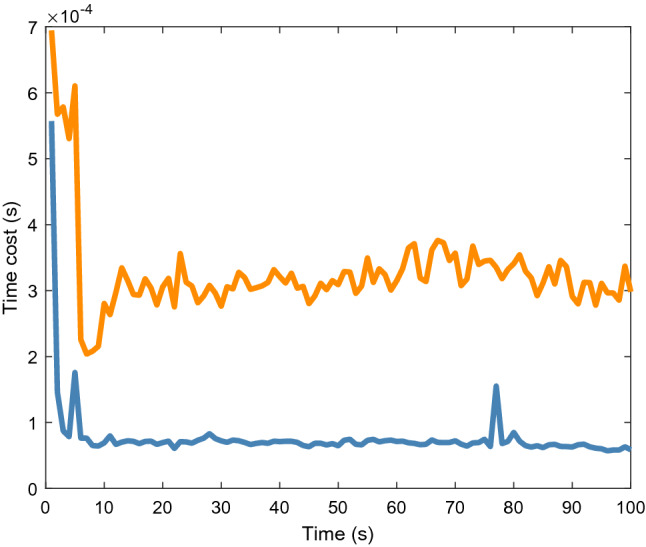
Average time cost of elliptical object.

Figure 6 shows the average time cost of 200 Monte Carlo runs. Although it can be observed that the proposed algorithm has a higher tracking accuracy, the time cost is approximately twice that of the original algorithm. Therefore, the proposed method is more suitable for 2D lidar systems that are insensitive to such cost.

### Rectangular object scenario

In this section, the target is rectangular. The shape of the vehicle is often rectangular in the real world; thus, the rectangular object can be used to test the performance of the proposed method for the tracking of non-elliptical objects.

**Figure 7:**
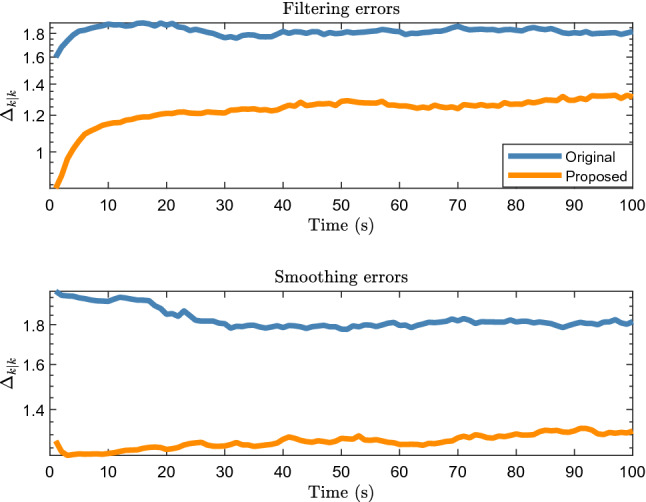
Average filtering and smoothing results of rectangular object.

Figure 7 shows the filtering and smoothing results of 200 Monte Carlo runs. The blue and red ellipses represent the results obtained using the original and proposed methods, respectively. The green rectangle indicates the vehicle location. The black square represents the location of the lidar. The proposed method still achieves a better performance.

**Figure 8:**
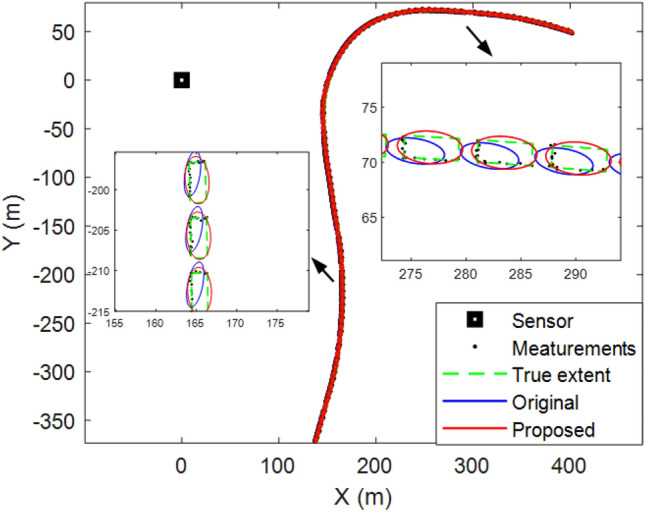
Rectangular object tracking results of a single run.

Figure 8 shows the results of a single run. It can be seen that although the shape of the object is rectangular, the proposed method can still use an ellipse to approximate its shape. Therefore, the proposed method is robust and can be applied to real scenarios such as vehicle tracking.

**Figure 9:**
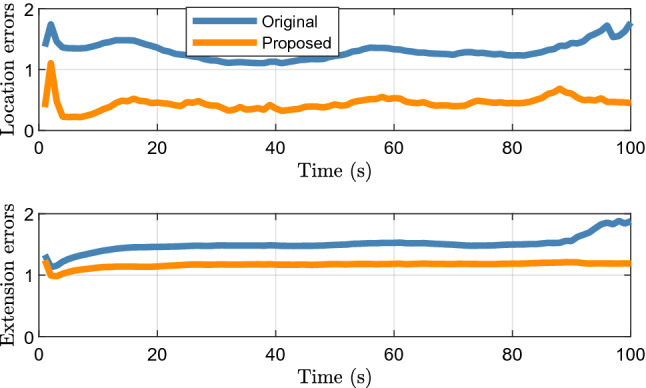
Average location and extension errors of rectangular object.

**Figure 10:**
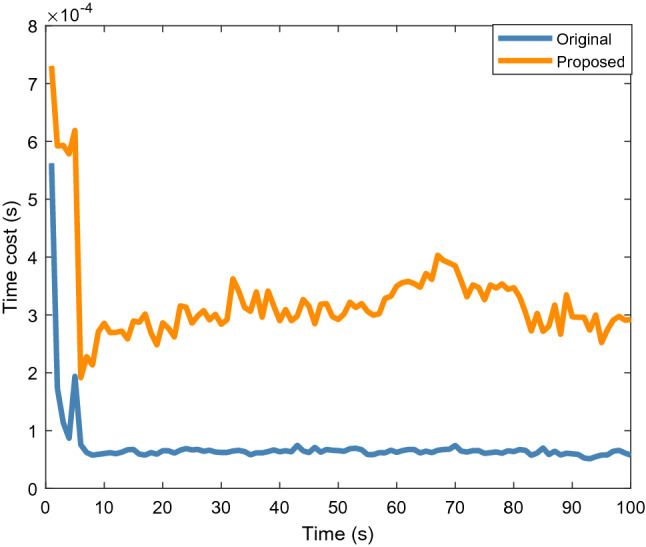
Average time cost of rectangular object.

Figure 9 shows the average location and extension errors of the rectangular object. It can be observed that the proposed method is still effective in reducing the location errors compared with the original filter when tracking a rectangular object. At the same time, the problem of a high time cost, shown in Fig. 10, still exists; thus, users should comprehensively consider the balance between accuracy and time cost.

### Real 2D lidar data scenario

**Figure 11:**
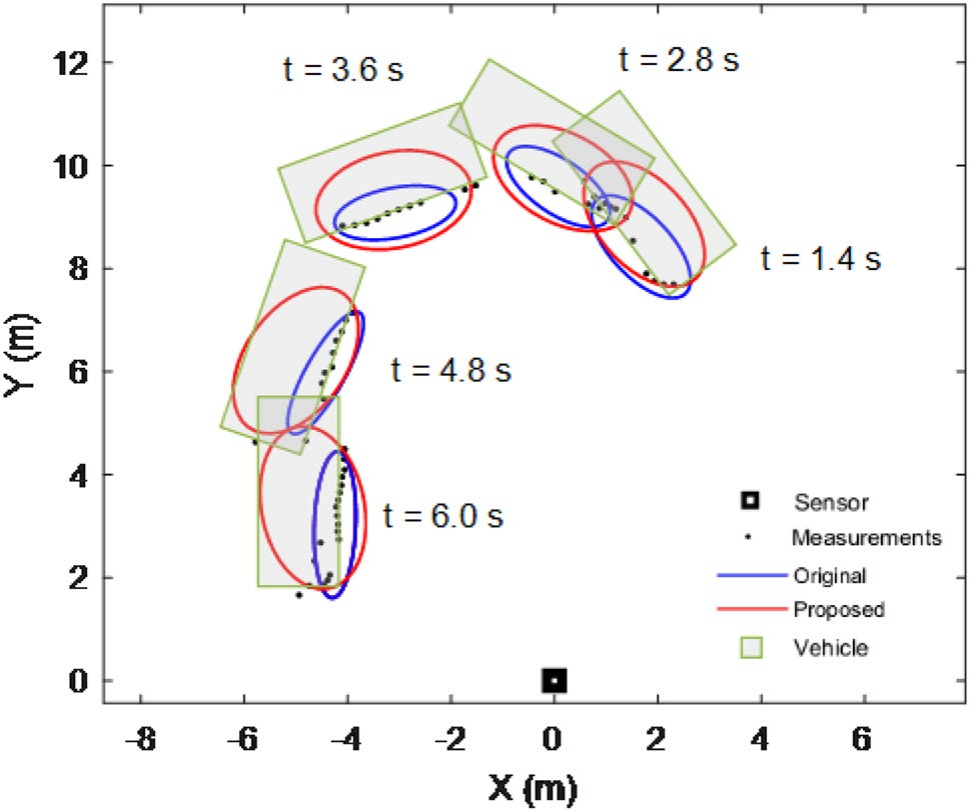
Tracking results of real 2D lidar data scenario.

In this section, real-world 2D Lidar data used to test the effectiveness of the proposed method are described. The object in the scenario was a car, and 60 data scans were obtained using 2D lidar. The vehicle turns left on the open ground (as shown in Fig. 11). Note that the location data of the vehicle comes from manual markings.

Figure 11 shows the tracking results of five scans. The green rectangle indicates the vehicle position. It can be observed that the tracking performance of the proposed method is better than that of the original approach.

**Figure 12:**
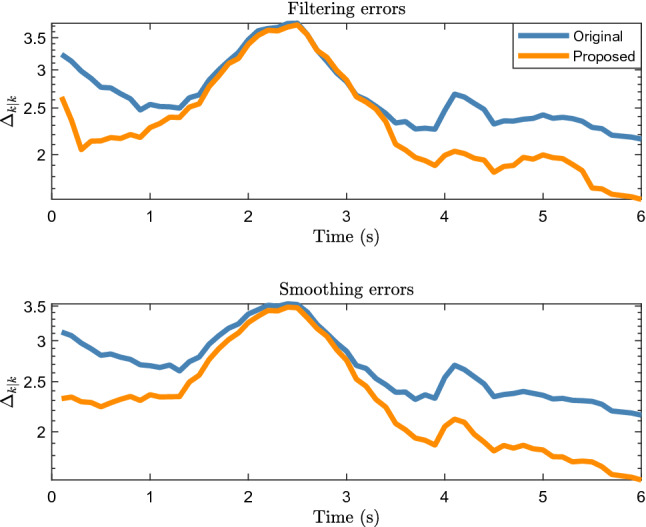
Filtering and smoothing results of real 2D lidar data scenario.

Figure 12 shows the filtering and smoothing results. It can be seen that the errors of both the proposed and original methods are large and similar at 1.5 to 3 s. According to Fig. 11, the car is turning during this period, and the lidar measurement is significantly affected by the environment and the inclination angle of the vehicle surface. According to Eq. (10), when the number of midpoints is relatively small, that is, $\overline{n} < \vartheta$, the proposed method becomes.

$$\hat{z}_{k} = \sum\limits_{i = 1}^{{n_{k} }} {\omega^{\left( i \right)} z_{k}^{\left( i \right)} }$$.

It is the weighted average of the measurement taking into account the influence of the object contour inclination and similar to the original RM method (see the Eq. (39) in^8^), which is why the accuracy of the proposed method is similar to that of the original method in 1.5 to 3 s. Therefore, the tracking accuracies of the proposed and original methods are significantly reduced. However, the accuracy of the proposed method is higher than that of the original method for other time periods, which shows that the proposed method is effective for use with real 2D LiDAR data.

**Figure 13:**
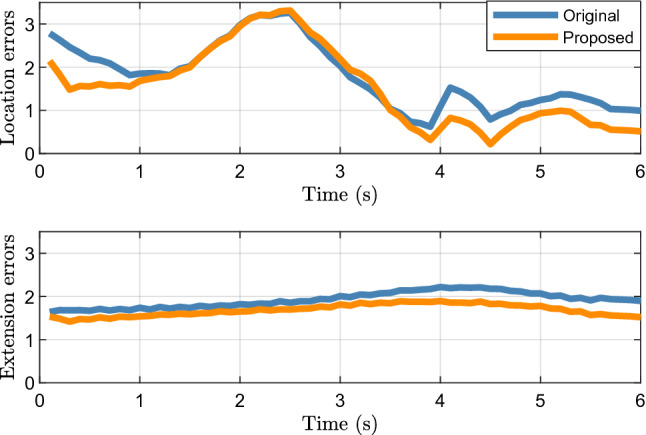
Location and extension errors of real 2D lidar data scenario.

**Figure 14:**
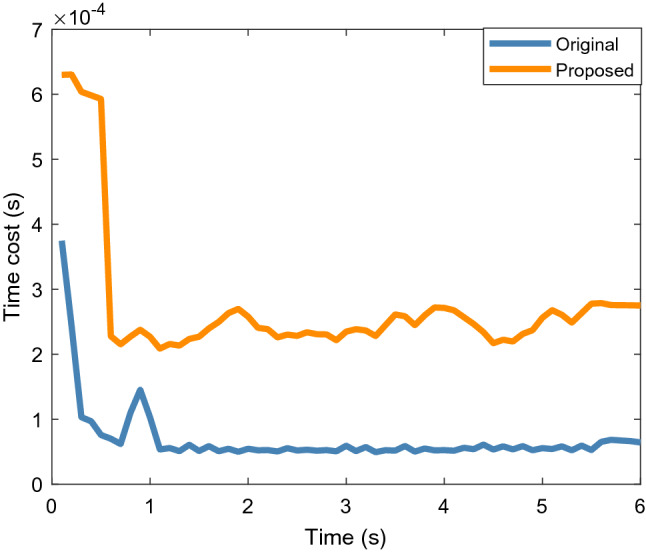
Time cost of real 2D lidar data scenario.

Figure 13 shows the location and extension errors. The location errors of both the proposed and original methods increase significantly within 1.5 to 3 s, whereas the expansion error fluctuates less during this period. This shows that the increases in the errors in Fig. 12 within 1.5 to 3 s are mainly caused by location errors. Figure 14 shows the time cost. Similar to the simulation scenarios, the time cost of the proposed method for processing real 2D Lidar data is higher than that of the original method.

## Conclusion

In this paper, a modified RM model was proposed for tracking an extended object using a 2D lidar system. Compared to the original model, the proposed method can better estimate the location and extended state of an object using the physical characteristics of the lidar system. The simulation results show lower GWD errors for the proposed method than for the original approach.

Although some RM-based tracking methods have also discussed the tracking problem in a lidar system, the tracking results should be calculated according to the characteristics of specific objects. For example, in^17^, information such as the front direction and steering angle of the front wheels was used; thus, the method is only suitable for the tracking of rectangular vehicles. The proposed method provides another interesting idea, i.e., a way to make full use of the measurement characteristics of a lidar system to estimate the real state of an object. Therefore, the proposed method is not limited to vehicle tracking. However, it should be noted that the method is only applicable to lidar systems with a high measurement accuracy. If the amount of noise is excessive, the results calculated by Eqs. (8) and (9) may have large errors, which is also a problem with the proposed approach.

In a future study, we plan to apply the proposed method to multiple extended object-tracking filters, such as a PMBM filter^12^.

## Data Availability

The data generated during this study are included in this article.
